# Medico-Chirurgical Transactions

**Published:** 1816-09

**Authors:** 


### *Medico-Chirurgical Transactions, Vol. vi.*

( Continued from page 146. )

*Statements of the comparative Health of the British Navy from the Year* 1779 *to the Year* 1814, *with Proposals for its farther Improvement.* By Sir Gilbert Blane, Bart. &c.

Prolix as is this paper, it needs not detain us long; since it is an historical record conveying no interest to the general medical reader. Besides, the gates of Janus are now closed, and the sun of the British navy is probably set for ever! Its last beams shone with an ominous, though resplendent lustre on the heights of Trafalgar; but they are now buried in the *Atlantic!*

Events, which no human sagacity could foresee, perhaps no human power controul, have altered the face of things. England can never be a great naval, and at the same time a great military nation. The *latter* she seems to have determined on, and naval hygiene may therefore be considered as an uninteresting and unimportant topic. The naval *medical* spirit is also broken. The corps is degraded in their own eyes, and in the eyes of the nation. They are not considered as at all fit to be placed on a par with their brethren in the army, but signally and pointedly placed beneath them in every respect of rank and emolument! All this, too, is done by those whose blood was stopped, whose wounds were healed, whose lives were preserved in every conflict with the enemy, or with the elements, round the circumference of the globe!

The various means by which the British navy attained the state of health in which it stood at the close of the war, compared with what it was at the period of the French revolution, are detailed by Sir Gilbert Blane; but he has omitted many, under-rated some, and over-valued others. The subject may probably form a separate article of communication by one of the Editors, whose experience and observations qualify him for the task.

We shall take notice here of Table No. 1 in Dr. Blane's paper, which requires some explanation.

### Comparative Mortality in Haslar and Plymouth Hospitals.

Haslar.

| | | | | | | |
|---|---|---|---|---|---|---|
| In 1779 | received | 15141 | — | died | 807 | or 1 in 18¾ |
| In 1782 | ditto | 9103 | — | ditto | 513 | or 1 in 17¾ |
| In 1794 | ditto | 8949 | — | ditto | 496 | or 1 in 17¾ |
| In 1804 | ditto | 1667 | — | ditto | 140 | or 1 in 12 |
| In 1813 | ditto | 3592 | — | ditto | 212 | or 1 in 16¾ |
| | Total | 38452 | | | 2168 | or 1 in 17½ |

Plymouth.

| | | | | | | |
|---|---|---|---|---|---|---|
| In 1779 | received | 6799 | — | died | 174 | or 1 in 39 |
| In 1782 | ditto | 4784 | — | ditto | 136 | or 1 in 35 |
| In 1794 | ditto | 4237 | — | ditto | 164 | or 1 in 26 |
| In 1804 | ditto | 3888 | — | ditto | 282 | or 1 in 13 |
| In 1813 | ditto | 3563 | — | ditto | 231 | or 1 in 15 |
| | Total | 21271 | | | 987 | or 1 in 21½ |

Now it must appear somewhat strange, that in two English hospitals on the same line of coast; receiving sick from the same fleet; both on the same establishment, and officered with a similar description of gentlemen, there should yet be such a disproportion of recoveries: not in one year only, but *in five years,* as that of 17 to 21. This cannot *apparently* be attributed to accident, because the parallel is drawn in the *same years* at each place; and consequently those unacquainted with the subject would very naturally be led to suspect, that there existed a striking difference of salubrity in the two situations, or some unaccountable difference of treatment pursued by the medical superintendants.

The medical topography of Haslar, however, and also the medical officers of that establishment, will bear a comparison with those of the Plymouth institution, without much danger of an imputation of inferiority. Sir Gilbert has not attempted an explanation of this phænomenon in the paper under consideration; but in his work on the Diseases of Seamen, he ascribes it to comparative insalubrity on the part of Haslar. On this point, however, we shall not enter, since *other* causes may be found that will sufficiently account for the circumstance.

In the first place, the Table shews, that during the three first years of the period, Haslar received considerably *more than double the number of sick* which the Plymouth institution received; and as fevers and other contagious diseases were *then* the order of the day, we may judge how unfavourable the circumstance of crowding alone was to the comparative mortality in Haslar. In the second place, the Table shews, and history proves, that Spithead was, in the years alluded to, the grand rendezvous of the channel fleet; and consequently the neighbouring hospital was the receptacle not only of the greater number of sick, but of the worst cases; so much so, that during these great influxes of disease, none but bad cases were sent to Haslar, while all slight ones were sent to Forton, Porchester, and various temporary or other hospital ships; and hence the mortality at Haslar.

In the third place, Portsmouth being the anchorage which was always made by ships returning from the East and West Indies, Africa, and almost every foreign climate, the miserable victims of these stations were annually, and we might say monthly, pouring into Haslar; a great majority of them only to drag out a short span of existence, before mingling with their mother earth.

During all this time, Plymouth was the rendezvous of detached ships, or cruising squadrons only, whose crews were always more healthy, and whose diseases were of a very different character from those of the channel fleet, in these periods of our naval history.

But when, in 1804, Lord St. Vincent threw the great body of ships on the Plymouth station, *then* we see that *there* they lost *one in thirteen* of their patients, which is far more than the medium loss at Haslar, notwithstanding its being the general receptacle of foreign diseases and their direful consequences.

To shew also, how very fallacious are *tabular* views of diseases, and how entirely the comparative mortality depends on the ever varying nature of the diseases admitted, we have only to compare Plymouth hospital *with itself*, at different periods of time: thus, in 1779, it received nearly 7000 patients, and only lost one in thirty-nine; but in 1804, when every thing under Lord St. Vincent was in *optimism* itself, Plymouth received little more than half the above number—yet lost *one in thirteen* of the patients!

We need not pursue the subject farther, as we think we have convinced the most sceptical, that hasty conclusions respecting the salubrity or insalubrity of a place should not be drawn from *figures* alone.

*Particulars of a Case in which a very large Calculus was removed from the Urethra of a Female without Operation, with Examples of analogous Cases.* By Dr. Yelloly, Physician to the London Hospital.

J. M. from the age of seven years, occasionally passed bloody urine, especially after considerable exertion in jumping. The general health was unimpaired, and no painful micturition was ever complained of. At twenty years of age she was married. About the seventh month of the first pregnancy, great difficulty in passing her water came on: this, however, disappeared on delivery. A purulent discharge, supposed to originate in gonorrhœa, attended the dysuria, and ceased at the same time with it.

In a second pregnancy, she was delivered of twins at seven months. Soon after this she became a third time pregnant; and, during the last three months of this pregnancy, the pain in making water, and the purulent-like discharge, returned: this latter symptom was, as before, referred to gonorrhœa.

The labour was favourable, but inability to retain her water supervened; and continual and urgent pain in the region of the bladder required the employment of large doses of laudanum. Though the discharge was diminished, a considerable deposit of a purulent appearance, as in the third pregnancy, was observed. Calculous concretions, of more than half an inch in length, were frequently passed, and gave temporary relief; and one oval stone, somewhat larger than these, was removed from the orifice of the urethra by a pair of forceps. At the same time, the presence of a large stone in the bladder was ascertained, both by sounding, and by the introduction of the finger into the vagina. Notwithstanding this, however, the enuresis still remained, and frequent and severe returns of pain were experienced.

In six months from this time, the advance of the stone into the urethra was perceptible to the patient; and Mr. Hopké, her medical attendant, in a few days could distinguish it from the orifice of the urethra, which was greatly dilated: no communication was discovered, on manual examination, between the urethra and vagina. This fact was confirmed by Mr. Headington, surgeon to the London Hospital, who, eight days after Mr. H's examination of the patient, removed the stone with ease, by means of his two fore fingers.

The calculus thus removed was irregular in surface, and in shape a flattened oval, having two small rounded projections on the end, by which it passed from the urethra. Its weight is 3 ounces $3\frac{1}{2}$ drachms troy; its length, $3\frac{1}{8}$ inches; its breadth, 2 inches; its thickness, $1\frac{7}{8}$ inch: its larger circumference, $7\frac{1}{4}$ inches; and its smaller one, $5\frac{1}{2}$ inches. Its chemical composition is said to be principally uric acid, disposed in concentric lamellæ, with a great portion of the surface covered with a mixture of phosphate of lime and ammoniaco magnesian phosphate.

A descriptive plate is annexed.

Though the patient's health was greatly improved on the removal of this calculus, and the catamenia returned after a long cessation (viz. from the time of the third pregnancy), yet she still continued to pass her urine involuntarily.

In another pregnancy, which took place two years afterwards, she suffered greatly from pressure of the child's head on the os pubis, according to her own account. During the latter months, she was harassed with cough and spitting, which were aggravated after delivery, though she had a favourable time. The lochial discharge was scanty, milk soon dried up, strength rapidly sunk, and she died on the 25th of December, three weeks after delivery, in the 28th year of her age. During the last fortnight of her life, she passed several sabulous concretions. The body was not examined, a circumstance to be greatly regretted; it would have been interesting to have examined the state of the urinary organs in one so long afflicted with calculous diseases, and likewise to have traced, if possible, the progress of the symptoms, as marked by the structural derangement, if such existed.

Why did the lungs suffer so much in this case? We are not told that the unhappy sufferer was at all predisposed to pulmonic diseases. Dr. Yelloly has collected, principally from the Phil. Transactions, many accounts of large calculi being removed from the female bladder, either by the spontaneous efforts of the body, or by slight dilatation and extraction with the forceps: the most remarkable is recorded by Dr. Molyneux. The patient was 60 years of age, and passed, without any assistance from art whatever, a stone resembling a flattened pear, $7\frac{3}{10}$ inches in circumference the longest way, $5\frac{1}{4}$ inches where it was largest, and weighing 2 oz. 2 drs. 1 scr. and 6 grs. troy. It was three months lodged in the urinary passage, during which time she suffered great pain, constant strangury, and a perpetual dropping of her water; which last symptom continued after the removal of the stone.

The Doctor concludes the paper with noticing, that in most cases of the same nature, as well as in that which he has laid before the Society, enuresis is present ever after the removal of the stone: this has been attributed to ulceration of the urethra, supposed to take place and to facilitate the passage of the stone through the vagina. It is pretty clear, that no such communication was established in this case; nor can we see that there is any necessary connexion between ulceration and enuresis. The irritation of an extraneous body so rough as these calculi in general are, is enough to cause a constant dribbling of water during its passage, and the mechanical injury induced by its weight and bulk sufficiently explains the inability to hold the water which remains so long after the calculus is removed. The vagina in the female, and the gall-ducts in both sexes, afford remarkable points of analogy on the subject of the dilatability of the urethra. Many more might be mentioned.

*Case of Mortification of the Uterus occurring a few Hours after Delivery.* By Thomas Graham, Esq.

A Lady, ætat. 37, had four times aborted between the 5th and 6th months. In the summer of 1812, she had slight paralysis of tongue and right arm. In the 6th month of utero-gestation of the 5th child, she complained greatly of the weight of the child, and want of power in the abdominal muscles to support it. The uterus appeared unusually large for the period of gestation. A wasting of the muscles, and a sudden obesity, accompanied the foregoing symptoms.

In the beginning of the 7th month, half a pint of fluid suddenly issued from between the chorion and amnion. On the following day, the os uteri dilated, and the membranes protruded; they were ruptured with difficulty, when *ten pints of water flowed off.* Feet presented, and a small sickly child was easily delivered, the placenta readily separating without difficulty. Except some sensation of emptiness and faintness, nothing unpromising occurred till next day, when a severe rigor of some minutes was succeeded by acute pain in the region of the uterus, with a great degree of heat extending to the groins. Venæsectio ad uncias ℥xvj. Six grains sub. hyd. with a scruple of rhubarb instantly. In two hours, pulse reduced; countenance pale and contracted; sensorium undisturbed. Pain continuing, a blister was applied to the abdomen, and sixty drops of tinct. of opium given in injection. Death in six hours from the rigor.

*Sectio cadaveris.* Abdominal muscles could scarcely be distinguished among so much fat; intestines distended with air; no effusion in the cavity of the abdomen. The uterus uncontracted, and of a dark and livid hue, with several gangrenous spots on the internal surface.

Mr. Graham thinks the two following causes may be assigned for this sphacelus of the uterus, viz.—1st. The accumulation of the liquor amnii producing, by its pressure, a morbid change in the structure of the uterus. 2d. The strength of the patient's constitution was unequal to the work of gestation after four successive abortions; the thickened state of the membranes retained the waters, and prevented an abortion this time at the usual period.

### *On the Use of Lactuca Virosa in Hooping Cough.* By Dr. Gumprecht, of Hamburgh.

Dr. G. considers that the disease above mentioned ought to be divided into the catarrhal and convulsive stages; that the first must be treated by antiphlogistics, and the second with antispasmodics. It is in the latter that he has found the *extractum lactucæ virosæ* useful, as also in the dyspnœa of hydrothorax, and spasmodic asthma in general.

Dr. Chaufepié, in his Letter to Dr. Gumprecht, says,

"We have all found this medicine to be a valuable and powerful specific in the above-mentioned disease. Its efficacy is great in the second stage, or the convulsive period of the disease. To children of two years old, I gave it three times a-day; at first, in doses of half a grain, with sugar alone, united with any of the other medicines usually employed. At this age, I have not administered it in doses of more than three-fourths of a grain. The coughing fits soon become more rare, and more gentle; the expectoration took place with less effort, and in general without vomiting; and the spasmodic affection in the region of the diaphragm apparently gave way. Upon the whole, this medicine seems, in a short time, to give the cough a different character, and considerably to diminish its duration."

Drs. Hempel and Jacobsen also speak highly of this remedy. It has considerable influence on the skin and urinary organs, besides a strong anodyne effect. In the dyspnœa of hydrothorax, Dr. G. asserts, that

" When all diuretic and anti-spasmodic means are found to fail, an alleviation of the dangerous symptoms, if not a cure of the disease, may almost always be experienced from the use of the *lactuca virosa.*"

We wish these assertions may prove true; but we have long been in the habit of distrusting the cures of diseases by *new* remedies.

*Some Experiments on the Chemical Nature of Chyle, with Observations.* By Dr. Marcet.

These experiments were made on the chyle of animals fed, some on animal, some on vegetable food, in order to ascertain, if possible, the chemical differences, if any.

We need not detail the processes, but the results, *viz.*

| | Specific gravity. | Solid matter in 1000 parts including saline. | Saline matter in 1000 parts. | Charcoal in 1000 parts. |
|---|---|---|---|---|
| Exp. 1. Chyle from animal food | | 70 | | |
| —— from vegetable food | | 48 | | |
| Exp. 2. Chyle from animal food | 1021 | 95 | 9.2 | 1 |
| —— from vegetable food | 1021 | 73 | 9.2 | 3 |
| Exp. 3. Chyle from animal food | 1022 | 74 | | |
| —— from vegetable food | 1022 | 78 | | |

Recapitulation.

The chyle from vegetable food appears to yield by analysis, about three times as much charcoal as that from animal food.

The chyle from animal food is infinitely more prone to putrefaction than that from vegetable.

*Further Observations on the Ligature of Arteries.* By Benjamin Travers, Esq.

It is now well known, that, if a ligature be instantly removed from an artery, after it is tied, the fissure in the middle and internal coats is soon obliterated by cicatrization, the vessel remaining pervious. But if three or four ligatures are applied contiguous to each other, or if one ligature be left on for some time, the vessel becomes blocked up by depositions of lymph, and by inflammation. The length of time necessary for the latter purpose is very uncertain, and is affected by difference of organic sensibility, or of temperament and vital power in different animals. The application of a ligature for six hours was almost invariably followed by obstruction of the artery, in those animals operated on by Mr. T.; but an indispensible condition is the division of the inner tunics.

To ascertain the earliest period at which the ligature might be removed, and the artery wounded (between it and another ligature an inch or two further from the heart) without hæmorrhage, was the object of the following experiments. These experiments we cannot detail, but we shall give some of the conclusions drawn from them.

1st. No material obstruction is opposed to the passage of the blood from six or even nine hours' ligature; the ultimate obstruction being the effect of the adhesive process.

2d. Six hours' residence of the ligature produces inflammatory action in the lymphy deposition between the divided tunics, which deposition is more abundant in nine hours, and sufficient for the obstruction of the vessel in twelve, presenting the form of an interstitial cord between the lips of the fissure, and continuous with it a membranous septum, extending across the vessel. This septum of lymph is, of itself, adequate to the prevention of hæmorrhage, but is liable to be ruptured under a sudden impulse of circulation, or violent concussion ab externo. The cylindrical coagulum of blood is an additional preventive to hæmorrhage, and is formed in twelve or twenty-four hours. A period of twelve hours is sufficient for the obstruction of the vessel by lymph, so as to admit of the removal of the ligature, and the wound or division of the artery without danger of hæmorrhage.

From Mr. Travers's interesting experiments on the effects of *compression* as a mean of obliterating the canal of an artery, we can only give the following deduction.

" It appears, then, that the operation of the compressor and ligature, applied each for one minute, is that of simple pressure opposed to simple wound. If they are applied for a longer time, it is simple pressure opposed to wound and pressure combined. It is undeniable, from these experiments, that the latter is a more powerful instrument for effecting the object in view; and this result of the combination is equally conformable to reason and to experience."

This article is very creditable to the well-known talents of its author.

We have now closed the sixth volume of the Medico-Chirurgical Transactions, and by means of our analytical *filter*, have presented our readers with a *depurated* stream of valuable information. It is curious to observe the different, often diametrically opposite employments of mankind—some building up hypotheses, others tearing them down:—some *expanding* facts into the utmost possible state of tenuity; others *condensing* them back again into their original form! By this alternate dilatation and contraction of the *central* organ in the medical body politic, the vital stream of knowledge is circulated to the periphery; otherwise, *accumulations* would certainly take place about the *head* and heart of the literary system; and while these important organs were *gorged*, or in a state of repletion, threatening *apoplexy* or suffocation, the consequent *inanition* of the extremities would cause paralysis or *mortification!* But to drop metaphor.—*Economy* is now a virtue which nine-tenths of the profession must practice *per force*; and it is only through the channels of *analyses*, that the knowledge of the upper classes of medical society can be diffused among the lower; and, consequently, that any thing like an equilibrium in medical literature can be preserved. When we say *economy*, we do not apply the word exclusively to *pecuniary* matters, but to *time* also. Many who could afford money to purchase most medical publications, cannot afford time to peruse them, except in garbled extracts from periodical Journals, that convey no clear or connected ideas of the originals. It is to remedy this defect that our analytical labours are directed. Time will shew the effect of our exertions.

